# Modified Measles in an Undervaccinated Adult Traveler

**DOI:** 10.4269/ajtmh.25-0593

**Published:** 2026-04-07

**Authors:** Chihaya Saito, Kazuhisa Yokota

**Affiliations:** Department of Infectious Diseases, Tokyo Bay Urayasu Ichikawa Medical Center, Urayasu, Japan

A 49-year-old man presented to the study hospital in May 2024 with a 1-week history of fever, sore throat, cough, and rash after returning to Japan from Vietnam, where a large measles outbreak was ongoing. He initially had a fever of 38.0°C, which resolved within 2 days, followed by a facial rash that extended to his trunk and upper limbs. Despite multiple clinic visits, no diagnosis was made. The day before his presentation, the fever recurred, reaching 38.7°C. He denied coryza, cough, or conjunctivitis. His vaccination history, based on patient self-report, indicated a single childhood dose of the measles vaccine administered around 1 year of age, consistent with Japan’s national one-dose measles schedule for children born before 1990.

**Figure 1.**
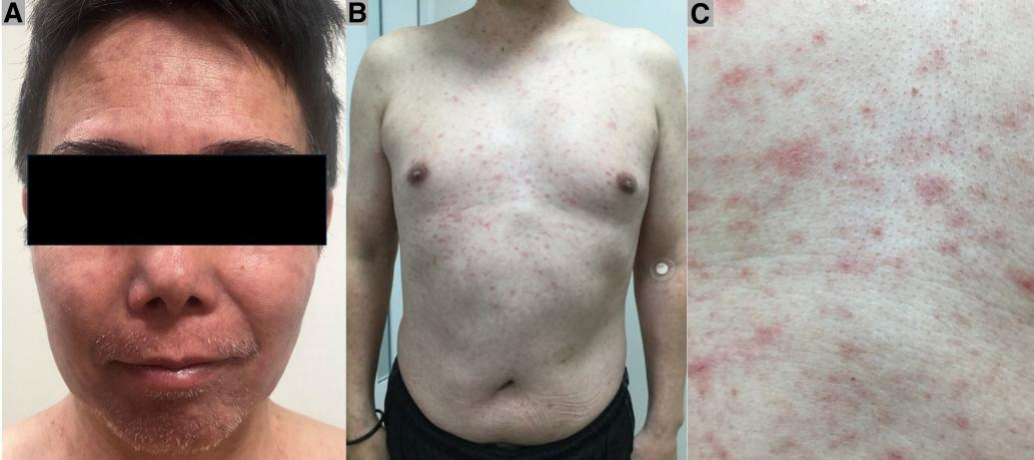
Maculopapular rash observed in a patient with modified measles. (**A**) Erythematous rash on the face. (**B**) Scattered maculopapular lesions on the anterior trunk. (**C**) Close-up view of the maculopapular rash on the anterior trunk.

On examination, scattered 5–10 mm maculopapular lesions with poorer confluence than typically observed in classic measles were noted (Figure 1). There was no cervical lymphadenopathy, and the buccal mucosa exhibited no Koplik spots. Blood testing revealed mildly elevated liver aminotransferase levels and atypical lymphocytosis. Chest X-ray findings were unremarkable. Serological assays revealed elevated anti-measles immunoglobulin M and immunoglobulin G, and measles virus was confirmed via polymerase chain reaction from a throat swab specimen (cycle threshold value: 31.2). The patient was instructed to isolate at home and recovered fully with supportive care.

Although measles is considered vaccine-preventable, infection may still occur in undervaccinated or, more rarely, fully vaccinated individuals. In such cases, symptoms tend to be milder and may lack the classic features of measles, such as high fever, cough, conjunctivitis, coryza, and a generalized confluent rash.^1^ This atypical presentation, which occurs in individuals with previous exposure or vaccination, is referred to as modified measles.^2^ The present case is an example of modified measles in an undervaccinated adult returning from an area with an ongoing outbreak.^3^ The patient lacked the typical respiratory prodrome and exhibited an unusual rash pattern, factors that likely delayed diagnosis. Because transmission from vaccinated patients is uncommon but possible,^4^ this case underscores the importance of obtaining a detailed travel history for timely recognition. Although the total number of close contacts could not be determined because of difficulty in identifying all possible exposures, one secondary measles case was confirmed through a public health investigation, highlighting that transmission can occur even from modified measles.
